# Prevalence of *Cryptosporidium* spp. in Yaks (*Bos grunniens*) in China: A Systematic Review and Meta-Analysis

**DOI:** 10.3389/fcimb.2021.770612

**Published:** 2021-10-18

**Authors:** Hong-Li Geng, Hong-Bo Ni, Jing-Hao Li, Jing Jiang, Wei Wang, Xin-Yu Wei, Yuan Zhang, He-Ting Sun

**Affiliations:** ^1^ College of Veterinary Medicine, Qingdao Agricultural University, Qingdao, China; ^2^ General Monitoring Station for Wildlife-Borne Infectious Diseases, State forestry and Grass Administration, Shenyang, China; ^3^ College of Life Sciences, Changchun Sci-Tech University, Shuangyang, China; ^4^ College of Animal Science and Veterinary Medicine, Heilongjiang Bayi Agricultural University, Daqing, China; ^5^ College of Animal Science and Technology, Jilin Agricultural University, Changchun, China

**Keywords:** *Cryptosporidium* spp., yaks, China, meta-analysis, prevalence, zoonosis

## Abstract

*Cryptosporidium* spp., the causative agent of cryptosporidiosis, can infect a variety of hosts. So far, there has been limited information regarding *Cryptosporidium* spp. infection in yaks (*Bos grunniens*). Here, we performed the first systematic review and meta-analysis for *Cryptosporidium* spp. infection in yaks in China. To perform the meta-analysis, five databases (Chinese National Knowledge Infrastructure (CNKI), VIP Chinese journal database, WanFang Data, PubMed, and ScienceDirect) were employed to search for studies related to the prevalence of *Cryptosporidium* spp. in yaks in China. The total number of samples was 8,212, and the pooled *Cryptosporidium* spp. prevalence in yaks was estimated to be 10.52% (1192/8012). The prevalence of *Cryptosporidium* spp. in yaks was 13.54% (1029/5277) and 4.49% (148/2132) in northwestern and southwestern China, respectively. In the sampling year subgroups, the prevalence before 2012 (19.79%; 650/2662) was significantly higher than that after 2012 (6.07%; 437/4476). The prevalence of *Cryptosporidium* spp. in cold seasons (20.55%; 188/794) was higher than that in warm seasons (4.83%; 41/1228). In the age subgroup, the yaks with age < 12 months had a higher prevalence (19.47%; 231/1761) than that in yaks with age ≥12 months (16.63%; 365/2268). Among 12 *Cryptosporidium* spp. species/genotypes, the *C. bovis* had the highest prevalence. Moreover, the effects of geography (latitude, longitude, precipitation, temperature, and altitude) and climate on *Cryptosporidium* spp. infection in yaks were evaluated. Through analyzing the risk factors correlated with the prevalence of *Cryptosporidium* spp., we recommend that effective management measures should be formulated according to the differences of different geographical factors, in order to prevent cryptosporidiosis and reduce economic losses in yaks in China.

## Introduction

*Cryptosporidium* spp. is an opportunistic protozoan that parasitizes the mucosal epithelial cells of gastrointestinal tract in animals (Wang et al., 2019a). *Cryptosporidium* spp. has a wide range of hosts, including cattle, cats, birds and human (Bhat et al., 2019). The transmission routes for *Cryptosporidium* spp. include a direct contact with infected animals, contaminated water or food, and fecal-oral route (Qin et al., 2014; Ryan et al., 2016; Yildirim et al., 2020). In general, the infection of *Cryptosporidium* spp. in individual was asymptomatic. However, severe symptoms may be induced in immunocompromised individual (Desai, 2020).

The average altitude of yaks’ (*Bos grunniens*) habitats is around 3,000 meters above sea level (Lan et al., 2020). The main habitats for yaks are in Tibet municipality, Qinghai Province, Gansu Province, and Sichuan Province (Wang et al., 2019b). Qinghai Province, which was identified to be the largest population of yaks in the world, has approximately 5 million yaks (Wang et al., 2018). So far, 38 species and over 70 genotypes of *Cryptosporidium* spp. have been identified (Deng et al., 2020). Twelve *Cryptosporidium* spp. species/genotypes have been identified in yaks, including *C. bovis*, *C*. *ryanae*, *C. baileyi*, *C. andersoni*, *C. parvum*, *C. hominis*, *C. canis*, *C. struthionis*, *C. xiaoi*, and *C. ubiquitum* (Ma et al., 2014b; Qi et al., 2015; Wang et al., 2018). More importantly, some of them, such as *C. parvum*, *C. hominis*, and *C. ubiquitum*, were also frequently found in humans (Widmer, 2009; Li et al., 2014; Ryan et al., 2016), and the infection rate is 36.4%, 9.3% and 1.6% (Guy et al., 2021). *Cryptosporidium* may cause fatal persistent diarrhea in infants and people with weakened or immune function and cognitive development, thus representing a public health threat (Xiao et al., 2004). The droppings of yaks that infected with *Cryptosporidium* spp. can be washed away by rain, thus resulting in an influx of *Cryptosporidium* spp. oocysts into the local source of water. The herdsmen and yaks, who live on the plateau, have a high probability to share the source of water. Thus, the yaks infected with *Cryptosporidium* spp. could bring the pathogen to herdsmen through the shared water (Wang et al., 2018). *Cryptosporidium* spp. infection in yaks can cause a loss of appetite, diarrhea, and other symptoms, which leads to a reduced resistance to the disease (Huang et al., 2014; Li et al., 2016a; Gong et al., 2017). The people living on the plateau can obtain various daily necessities (e.g., milk and beef) from yaks. Thus, the yaks are one of the important economic resources for the local people, leading to a direct correlation of yak’s health and economy (Mi et al., 2013). So far, there has been no effective drugs or available vaccines for preventing and controlling cryptosporidiosis (Gao, 2012; Ikiroma and Polloc, 2021). The prevention of cryptosporidiosis is an important approach for reducing losses to the breeding industry.

Currently, a systematic evaluation and analysis for cryptosporidiosis in yaks is absent. Thus, it is essential to carry out a systematic evaluation and meta-analysis based on the existing literatures. In this study, our study aim was to analyze the epidemic status of cryptosporidiosis among yaks in China, evaluate and discuss the corresponding risk factors that contribute to *Cryptosporidium* spp. infection in yaks.

## Methods

### Systematic Search Strategy

This paper was prepared according to the PRISMA guidelines for the design and analysis of selected qualified studies (**Table S1**). A literature search was conducted to identify articles published from the inception to January 18, 2021. The aim was to obtain all articles in Chinese and English with topics of *Cryptosporidium* spp. infection in yaks in China. The articles were collected from five databases, including China National Knowledge Infrastructure (CNKI), VIP Chinese Journals Database, Wanfang Data, PubMed, and ScienceDirect. The keywords “yak” and “*Cryptosporidium*” were used for searching on the databases CNKI, VIP Chinese Journals Database, Wanfang Data, and ScienceDirect. The MeSH terms “*Cryptosporidium*”, “yak” and “China”, and their entry terms, such as “*Bos indicus*”, “Zebu”, “*Bos taurus*”, “Domestic Cow”, “Domestic Cows”, “*Bos grunniens*”, and “*Cryptosporidium*” were used for searching on PubMed. The boolean operators “AND” and “OR” were used to connect MeSH terms and the entry terms, respectively. Finally, the search formula “((*Cryptosporidium*) OR *Cryptosporidiums*) AND ((((((((((((yak) OR *Bos indicus*) OR Zebu) OR Zebus) OR *Bos taurus*) OR Cow, Domestic) OR Cows, Domestic) OR Domestic Cow) OR Domestic Cows) OR *Bos grunniens*) OR Yak) OR Yaks)))) AND ((((((China) OR People’s Republic of China) OR Mainland China) OR Manchuria) OR Sinkiang) OR Inner Mongolia)” was used for searching on PubMed. The Endnote (X9.2 version) was employed to collate information of obtained articles.

### Data Extraction and Exclusions

The inclusion criteria for our systematic review and meta-analysis were as follows: (1) the subjects of the study were limited to yaks; (2) the detection of *Cryptosporidium* spp. was at least carried out by nucleic acid or pathogen detection methods, such as PCR, ELISA or microscopy; (3) the selected articles should contain the information of sample number, positive number, and detection site; (4) the article should contain a full-text with complete data; (5) studies must be designed for a cross-sectional extension; (6) the sample should come from a separate yak (not a mixed sample).

The extracted data included the first author, the year of publication, the province where the study performed, sample collection time, age and gender of yak, detection method, sampling seasons, geographical location (latitude and longitude), relative humidity, annual average temperature, annual precipitation, method type, total number of samples, number of positive samples, and data score. According to the report by Fan and colleagues, the climate of China’s plateau is unique, with the warm weather from June to October and the cold weather from November to May (Fan et al., 2011). Therefore, this division method was used to classify seasonal subgroups in this study. Our database was constructed by using Microsoft Excel (version 16.32). Two reviewers independently extracted and recorded data from each selected research. The differences derived from reviewers or uncertainty about the qualifications of the research were further assessed by another author of this paper.

### Quality Assessment

The standardized data collection table was used for data extraction according to the research purpose and inclusion criteria. The article quality was evaluated based on the Grading of Recommendations Assessment reported previously (Guyatt et al., 2008). The scoring criteria of data scoring items were as follows: (1) there was a detailed sampling time-point; (2) there was a specific sampling location; (3) the number of samples was over than 200; and (4) there were more than three risk factors. According to the above scoring criteria, 1 point was given for each item, and the total score of each item was added up to get the total score of the article. The total score was identified to be high quality for 3-4 points, medium quality for 2 points, and low quality for 0-1 points.

### Statistical Analyses

The meta package in R software version 4.0.3 (“R core team, R: A language and environment for statistical computing” R core team 2018) was used to analyze the data in this study (Li et al., 2020a). The W-value close to 1 and the *P*-value greater than 0.05 is identified to be close to the Gaussian distribution criterion. The double-arcsine transformation (PFT) method was chosen for data conversion (**Table 1**). The heterogeneity among studies was predicted by Cochran’s Q-value (represented by *X^2^* and *P*-value) and *I^2^* statistics. Cochran’s Q (*X^2^* and *P*-value) and *I^2^* statistics were employed to predict the inter-study heterogeneity. The random effect model was chosen for an analysis, according to the heterogeneity of the included articles (Ni et al., 2020). Forest plots were used for a comprehensive analysis. Funnel plot and Egger’s test were used to evaluate the publication bias. The stability of the study was evaluated by the trim and filling test, and sensitivity analysis (Wang et al., 2020).

**Table 1:** Normal distribution test for the normal rate and the different conversion of the normal rate.

Conversion form	W	*P*
PRAW	0.86549	0.009798
PLN	NaN	NA*
PLOGIT	NaN	NA*
PAS	0.93191	0.1681
PFT	0.93356	0.1808

“PRAW”: original rate; “PLN”: logarithmic conversion; “PLOGIT”: logit transformation;

“PAS”: arcsine transformation; “PFT”: double-arcsine transformation;

“NaN”: meaningless number; “NA*”: missing data.

The potential sources of heterogeneity were further studied by subgroup analysis and meta regression analysis. The individual and multivariate model factors were analyzed to determine the factors contributing to the heterogeneity. The survey factors included the sampling year (before 2012 *vs*. after 2012), region (Northwestern China *vs*. Southwestern China), province (Qinghai province *vs*. other provinces), diagnostic method (Immunofluorescence technique (IFA) *vs*. other methods), age (age < 12 months *vs*. age ≥ 12 months), season (cold seasons vs. warm seasons), genotype (*Cryptosporidium bovis vs*. other genotypes), the quality level of the included publications (high quality *vs*. others), longitude (95-100° *vs*. others), latitude (> 35° *vs*. others), altitude (< 95° *vs*. others), average annual precipitation (< 300 mm *vs*. > 300 mm), average annual temperature (< 1°C *vs*. others), average annual humidity (< 55% *vs*. ≥ 55%), altitude (< 3000 m *vs*. > 3000 m), and climate (plateau mountain vs. others).

## Results

### Search Results

**Figure 1:**
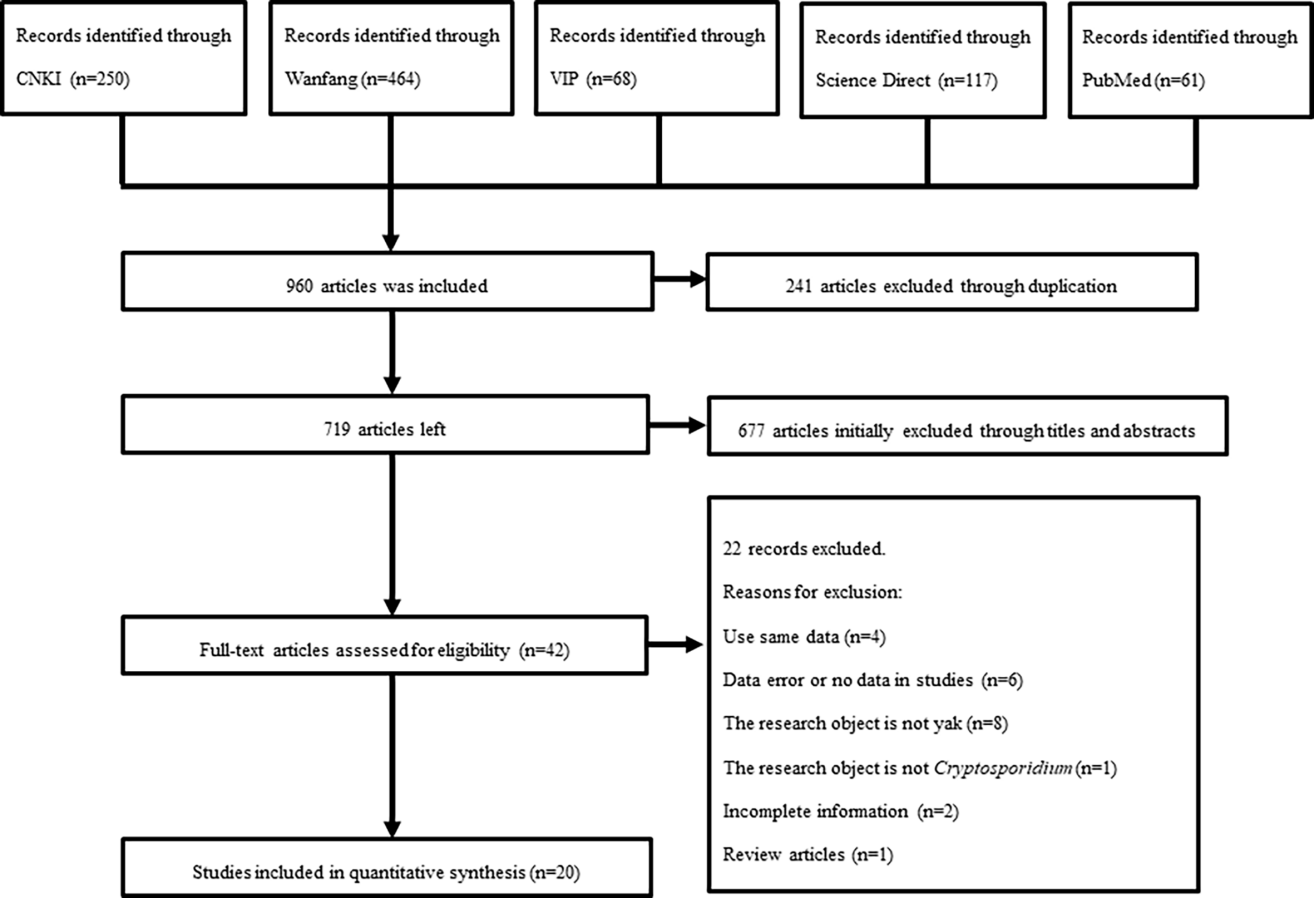
Flow diagram of literature search and selection.

Through searching on five databases, 1,006 relevant articles were screened out for further analyses. According to the selection criteria described in section “2.2”, the uncertain articles were excluded by checking the abstracts and/or full-text. Finally, 49 out of 1,006 articles were selected. Among of the selected articles, four were repeated publications, ten were not research objects, one was overview article and letter, and fourteen were removed due to an incomplete or unclear information. Thus, a total of 20 articles were included in this study (**Figure 1**).

### Qualification Studies and Publication Bias

Consequently, the included articles covered four provinces. Among the 20 studies, the total number of samples and positive number was 8,212 and 1,192, respectively (**Table 2**). Based on the quality standard, fourteen articles were of high quality (3 or 4 points), five were of medium quality (2 points), and one was of low quality (1 point; **Table 2** and **Table S1**).

**Table 2:** Pooled prevalence o*f Cryptosporidium* infection in yaks in China.

Variable	Category	No. studies	No. examined	No. positive	% (95% CI*)	Heterogeneity			Univariate meta-regression	
						*χ2*	*P*-value	*I^2^* (%)	*P*-value*	Coefficient (95% CI)
Season	Cold	5	794	188	20.55% (9.76–33.96)	63.24	< 0.01	95.3	0.012	0.331 (0.074 to 0.588)
	Warm	5	1228	41	4.83% (0.12-14.52)	0.14	< 0.01	0.0		
Age	<12 months	11	1761	231	19.47% (10.25–30.56)	241.34	< 0.01	95.9	0.659	0.042 (-0.145 to 0.229)
	≥12 months	6	2268	365	16.63% (7.84-27.84)	191.62	< 0.01	97.4		
Collection	Before 2012	7	2662	650	19.79% (9.34–32.83)	290.71	< 0.01	97.9	0.025	0.214 (0.027 to 0.400)
	After 2012	10	4476	437	6.07% (1.64–12.91)	540.89	< 0.01	98.3		
Method	PCR	12	5443	632	8.80% (3.83–15.45)	588.43	< 0.01	98.1	0.440	-0.088 (-0.312 to 0.136)
	Microscopy	7	1884	206	12.00% (4.04–23.29)	248.58	< 0.01	97.6		
	ELISA	2	1229	369	12.44% (0.00–58.23)	127.99	< 0.01	99.2		
	IFA	3	560	45	6.52% (0.16-18.65)	24.98	< 0.01	92.0		
Region*	Northwestern	15	5277	1029	13.54% (7.10–21.58)	826.49	< 0.01	98.3	0.093	0.160 (-0.026 to 0.346)
	Southwestern	6	2132	148	4.49% (0.60–11.22)	139.93	< 0.01	96.4		
Quality* level	High	14	6951	1056	12.14% (6.00-20.02)	1041.69	< 0.01	98.8	0.426	0.081(-0.118 to 0.280)
	Middle	5	859	123	7.76% (0.16-22.61)	119.43	< 0.01	96.7		
	Low	1	402	13	3.23% (1.70–5.22)	0.00	< 0.01	NA*		
Total		20	8212	1192	10.52% (5.64–16.63)					

CI*, Confidence interval; NA*, not applicable; P-value*, P < 0.05 is statistically significant.

Region*: Northwestern China: Qinghai, Gansu; Southwestern China: Sichuan, Tibet.

Quality*：High: 4 or 3 points; Middle: 2 points; Low: 1 point.

**Figure 2:**
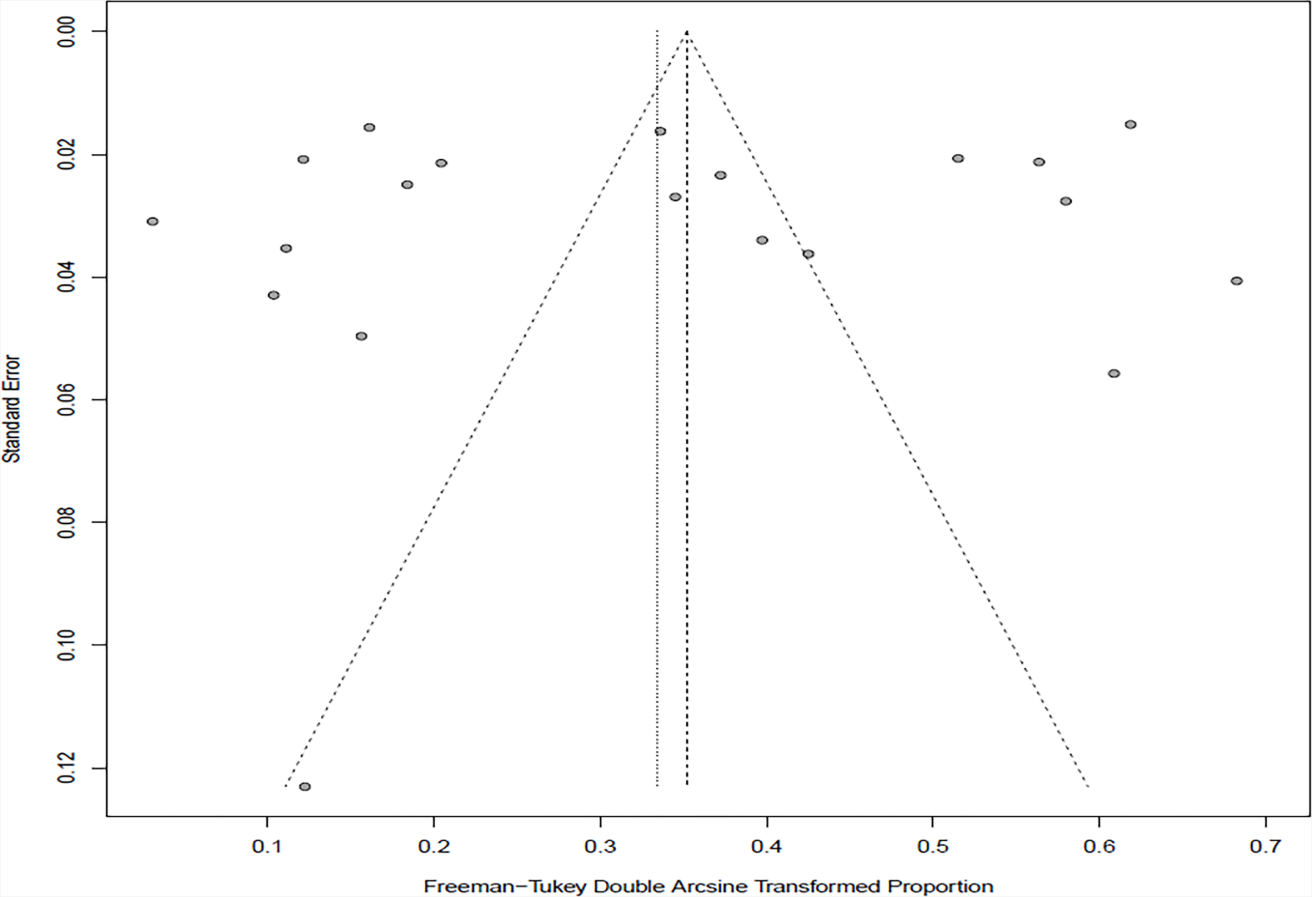
Funnel plot with pseudo 95% confidence interval for publication bias test.

**Figure 3:**
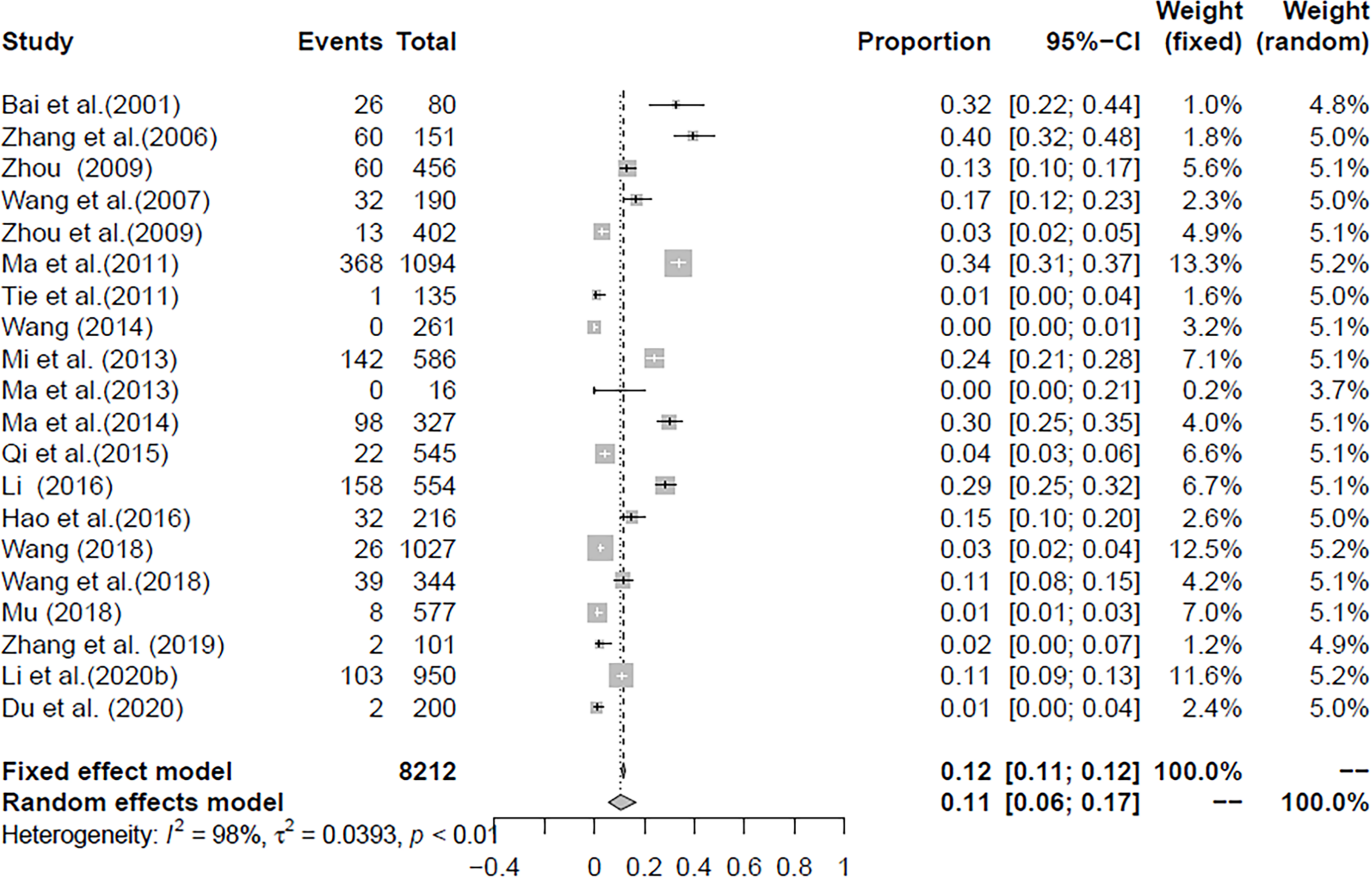
Forest plot of *Cryptosporidium* prevalence in yaks in China.

**Figure 4:**
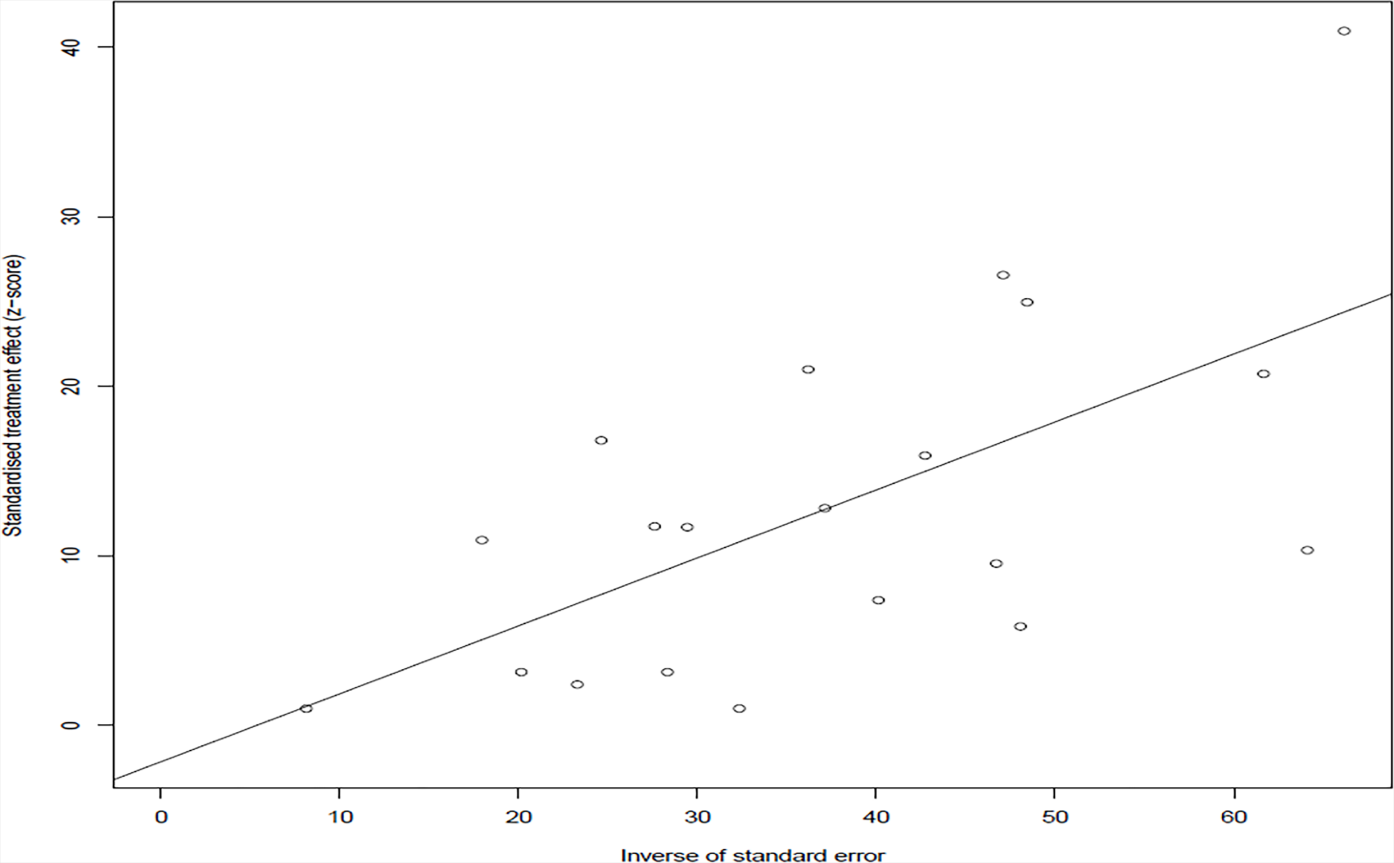
Publication bias of included studies by Egger’ test.

**Figure 5:**
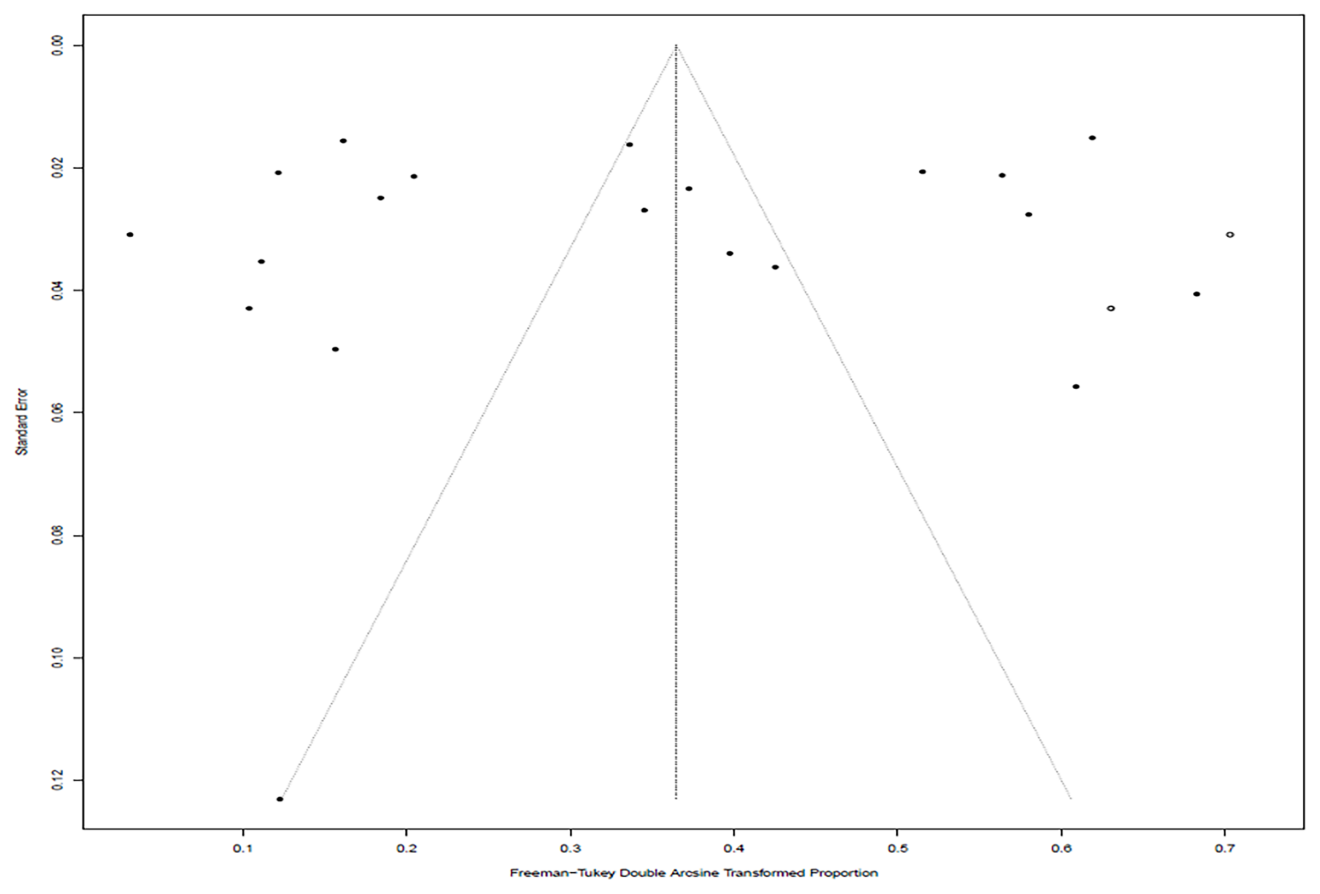
The trim and filling test.

**Figure 6:**
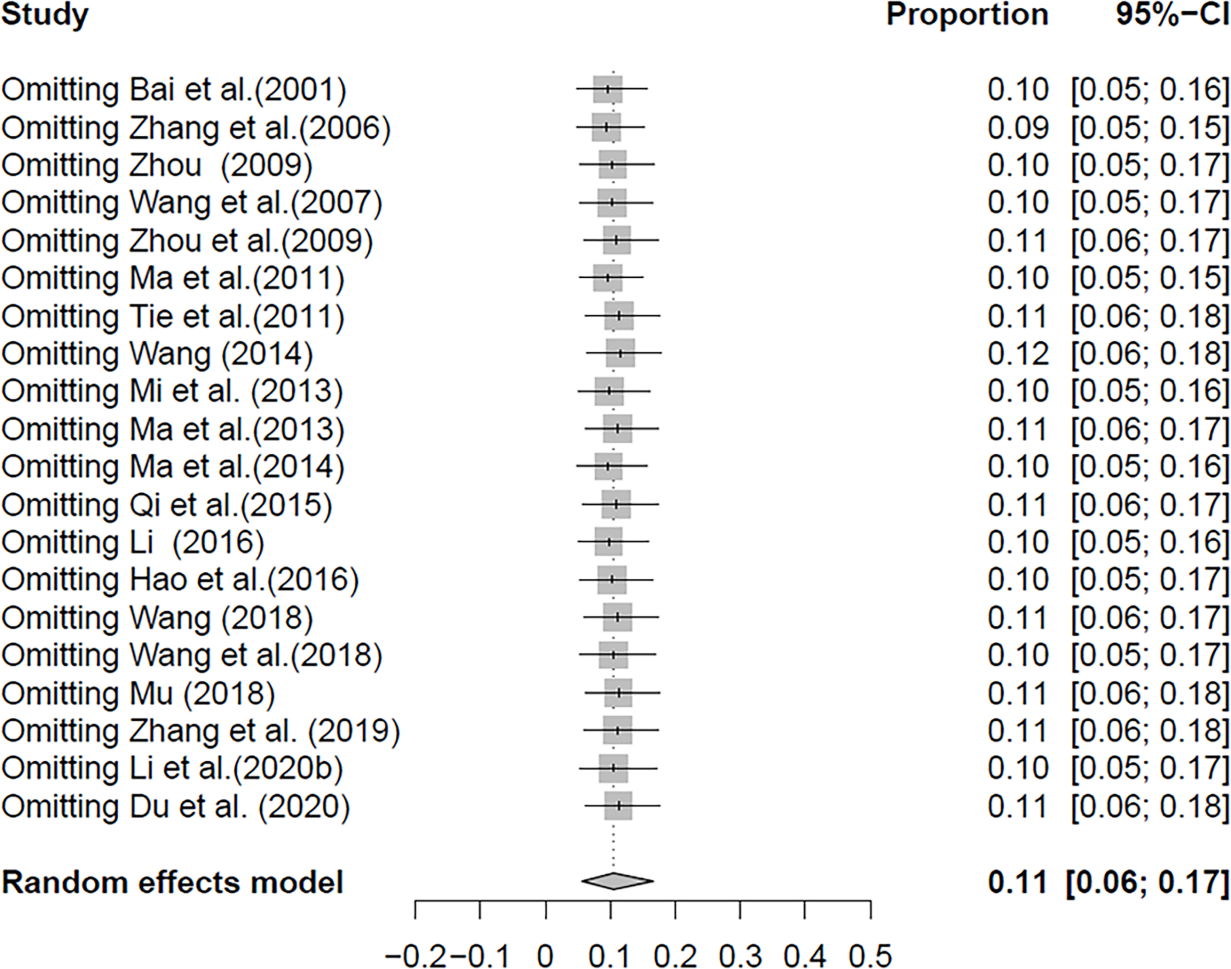
Sensitivity test.

In the selected studies, the forest plot measurement demonstrated the degree of heterogeneity (**Figure 2**). According to the funnel chart, we found that the distribution of dots was not completely symmetrical, which might be explained by publication bias or small sample bias (**Figures 3**, **4**). No supplementary study was found by the trim and filling test. The Egger test was used to assess the potential publication bias in the analysis, and the *P*-value greater than 0.05 indicated that no publication bias was present in the data (**Figure 5**). Sensitivity test indicated that the recombined data were not significantly affected by any study that was excluded (**Figure 6** **)**. These results verified rationality and reliability of our analyses.

### Results of the Meta-Analysis

From 2001 to 2021, the total prevalence of *Cryptosporidium* spp. in yaks in China was 10.52% (95% CI: 5.64-16.63; **Table 2** **)**. In region group, the higher prevalence was detected in northwestern China (13.54%, 95% CI: 7.10-21.58); than southwestern China (**Table 2**). In the covered four provinces of the meta-analysis, Qinghai province had the highest prevalence of 14.17% (95% CI: 7.34-22.70), and Gansu province and Tibet municipality had the lowest prevalence of 5.98% (95% CI: 2.29-11.12) and 6.03% (95% CI: 4.56-21.34), respectively (**Table 3**). To further identify sources of heterogeneity, we analyzed subgroups of season, age, sampling year, detection methods, detailed geographic, and climatic factors. Sampling year was a risk factor for *Cryptosporidium* spp. infection in yaks (*P* < 0.05; **Table 2**). The prevalence of *Cryptosporidium* spp. in yaks before 2012 was 19.47% (95% CI: 10.25-30.56), and 6.07% (95% CI: 1.64-12.91) in yaks after 2012 respectively (**Table 2**). Among 12 *Cryptosporidium* spp. species/genotypes, the *C. bovis* has the highest prevalence (0.34%, 173/4277, 95% CI: 0.16-0.25), followed by *C. andersoni* (0.25%, 100/4277, 95% CI: 0.19-0.32) and *C. parvum* (0.25%, 95% CI: 0.17-0.35; **Table 4**). The prevalence of *C. baileyi*, C. *ubiquitum*, and *C. xiaoi* were the lowest (0.02%, 1/4277, 95%CI: 0.00-0.40; 2/4277, 95%CI: 0.00-0.10; 1/4277, 95%CI: 0.00-0.10; **Table 4**). The information for subgroups analysis of geographical latitude included latitude range (> 35°; 14.68%, 95% CI: 6.19-25.85), longitude range (< 95°; 6.03%, 95% CI: 0.37-16.76), precipitation range (> 300 mm; 12.27%, 95% CI: 6.66-19.24), temperature range (< 1°C; 19.96%, 95% CI: 10.49-31.38), humidity range (≥ 55%; 12.36%, 95% CI: 4.74-22.80), and altitude range (< 3000 m; 13.15%, 95% CI: 5.38-23.60; **Table 5**).

**Table 3:** Pooled *Cryptosporidium* prevalence in yaks in various provinces.

Provinces	Regions	No. Studies	No. tested	No. positive	Prevalence (%)	95% CI
Qinghai	Northwestern	14	5160	1022	14.17%	7.34-22.70
Sichuan	Southwestern	3	561	33	3.15%	0.00-17.38
Tibet	Southwestern	3	1571	115	6.03%	4.56-21.34
Gansu	Northwestern	1	117	7	5.98%	2.29-11.12

**Table 4:** The species/genotype of *Cryptosporidium* in yaks was detected by PCR.

Category	No. studies	No. examined	No. positive	% (95% CI*)	Heterogeneity			Univariate meta-regression	
					*χ2*	*P*-value	*I^2^* (%)	*P*-value*	Coefficient (95% CI)
*C. ryanae*	10	4277	117	0.20% (0.16-0.25)	120.12	< 0.01	92.5	0.104	0.0183 (-0.0037 to 0.0403)
*C. bovis*	9	4277	173	0.34% (0.28-0.40)	173.36	< 0.01	95.4		
*C. baileyi*	1	4277	1	0.02% (0.00-0.10)	0.00	< 0.01	NA*		
*C. andersoni*	6	4277	100	0.25% (0.19-0.32)	144.65	< 0.01	96.5		
*C. suis-like*	1	4277	2	0.05% (0.00-0.14)	0.00	< 0.01	NA*		
*C. parvum*	3	4277	34	0.25% (0.17-0.35)	6.99	< 0.01	71.4		
*C. hominis*	1	4277	4	0.09% (0.02-0.21)	0.00	< 0.01	NA*		
*C. canis*	1	4277	3	0.07% (0.01-0.18)	0.00	< 0.01	NA*		
*C. struthionis*	1	4277	5	0.12% (0.03-0.25)	0.00	< 0.01	NA*		
*C. ubiquitum*	2	4277	2	0.02% (0.00-0.10)	0.00	< 0.01	0.0		
*C. xiaoi*	1	4277	1	0.02% (0.00-0.10)	0.00	< 0.01	NA*		
*C. new genotype*	1	4277	2	0.05% (0.00-0.14)	0.00	< 0.01	NA*		

CI*, Confidence interval; NA*, not applicable; P-value*, P < 0.05 is statistically significant.

**Table 5:** Sub-group analysis of the prevalence of *Cryptosporidium* according to geographic location and climate variables.

Variable	Category	No. studies	No. examined	No. positive	% (95% CI*)	Heterogeneity			Univariate meta-regression	
						*χ2*	*P*-value	*I^2^* (%)	*P*-value*	Coefficient (95% CI)
Latitude	< 30°	3	822	73	10.88% (0.00-39.41)	129.15	< 0.01	98.5	0.576	0.049 (-0.122 to 0.220)
	30-35°	9	2858	354	11.78% (5.62-19.77)	265.95	< 0.01	97.0		
	> 35°	12	3521	681	14.68% (6.19-25.85)	700.44	< 0.01	98.4		
Longitude	< 95°	3	1571	115	6.03% (0.37–16.76)	66.98	< 0.01	97.0	0.356	-0.126 (-0.393 to -0.142)
	95-100°	7	1094	198	14.22% (4.24–28.38)	185.29	< 0.01	96.8		
	> 100°	13	4536	795	13.44% (5.91–23.35)	865.07	< 0.01	98.6		
Precipitation (mm)	< 300	5	445	56	11.48% (6.00-18.27)	12.96	< 0.01	69.1	0.934	-0.007 (-0.182 to 0.167)
	> 300	20	6700	1054	12.27% (6.66-19.24)	1168.48	< 0.01	98.4		
Temperature (°C)	< 1	8	1759	292	19.96% (10.49–31.38)	147.67	< 0.01	95.3	0.178	0.125 (-0.057 to 0.307)
	1-5	11	2565	414	11.09% (4.17-20.58)	418.38	< 0.01	97.6		
	> 5	10	2659	399	11.71% (3.60-23.50)	538.62	< 0.01	98.5		
Humidity	< 55%	12	2868	365	11.09% (6.31-16.94)	194.38	< 0.01	94.3	0.825	-0.019 (-0.187 to 0.149)
	≥ 55%	13	3539	565	12.36% (4.74-22.80)	191.57	< 0.01	95.3		
Altitude (0.1 m)	< 30000	12	3646	677	13.15% (5.38–23.60)	806.97	< 0.01	98.5	0.850	-0.019 (-0.219 to 0.181)
	> 30000	16	3555	433	10.40% (5.32–16.83)	425.07	< 0.01	96.5		
Climate	Plateau mountain climate	18	6995	1158	14.02% (8.15–21.11)	1018.27	< 0.01	98.3	0.055	0.193 (-0.004 to 0.390)
	Temperate continental climate	1	117	7	5.98% (2.29–11.12)	0.00	< 0.01	NA*		
	Subtropical monsoon climate	4	211	12	2.77% (0.00-12.97)	65.08	< 0.01	95.4		

CI*, Confidence interval; NA*, not applicable; P-value*, P < 0.05 is statistically significant.

## Discussion

*Cryptosporidium* spp. can cause economic losses in animal husbandry, and bring a great threat to human health (Ouakli et al., 2018; Pumipuntu and Pirate, 2018). Therefore, it is essential to understand the prevalence of *Cryptosporidium* spp. in its hosts. A systematic review and meta-analysis of *Cryptosporidium* spp. prevalence among yaks in China was performed in this study. In 2012 and 2013, China issued the mid to long term animal disease prevention plan (2012-2020) and the National Development Plan for Beef and Mutton Production (2013-2020) to strengthen the prevention and control for animal diseases (Gong et al., 2020; Wei et al., 2021). Therefore, the year “2012” is taken as the cut-off time-point. After an introduction of the above policies, the effective prevention and control measurements might be one reason for the decreased prevalence of *Cryptosporidium* spp. after 2012 (General Office of the State Council, 2012).

**Figure 7:**
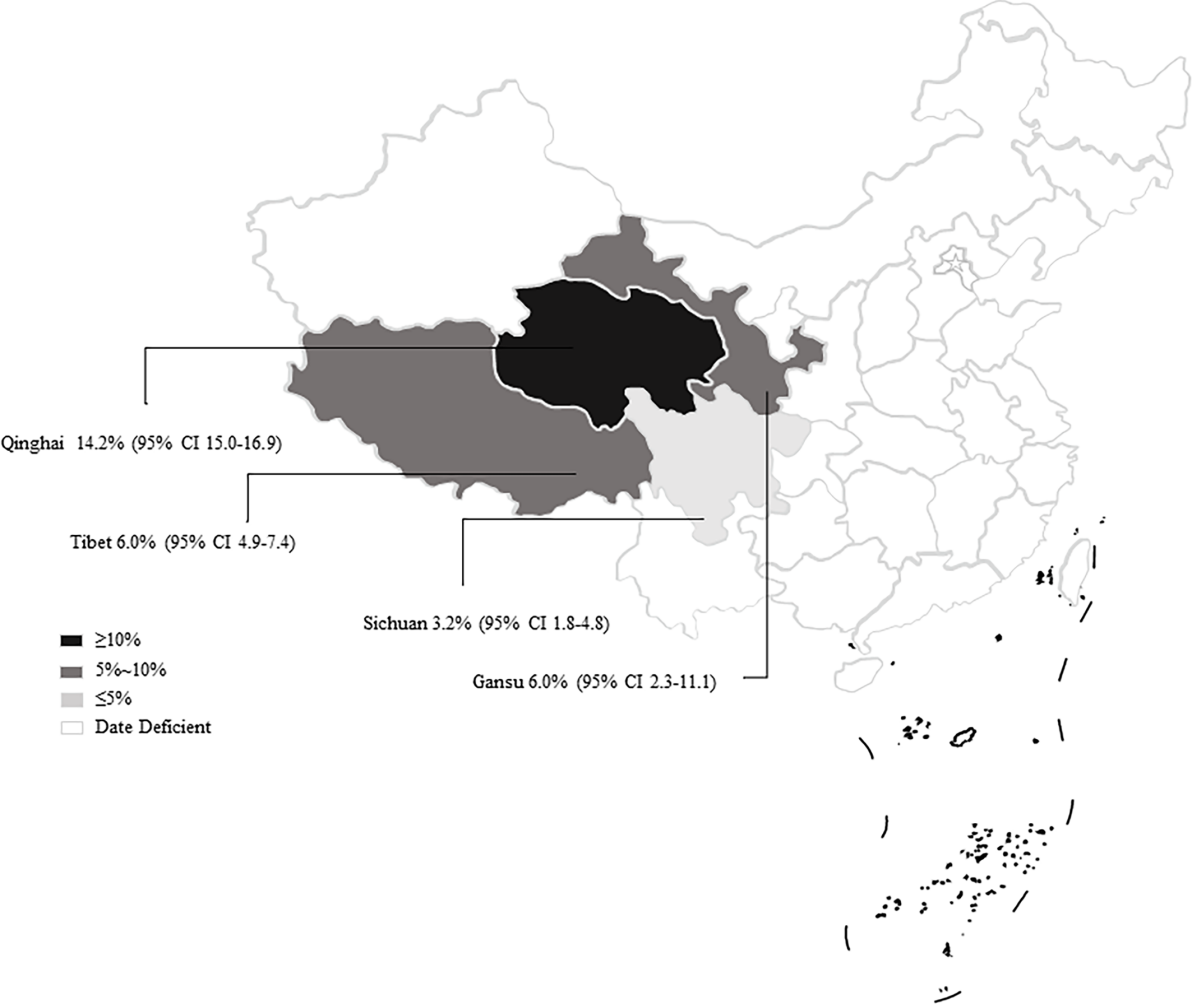
Map of *Cryptosporidium* prevalence in yaks in China.

In general, *Cryptosporidium* spp. prefers to live in a warm and humid environment, such as southwestern regions (Jagai et al., 2009; Taghipour et al., 2020). However, the prevalence of *Cryptosporidium* spp. in the northwestern regions was reported to be higher than that in the southwestern regions. We found that most of the articles retrieved in the southwestern regions were from Qinghai province (**Figure 7**). Qinghai province had a significant effect on the results of northwestern China. Meanwhile, the infection rate of *Cryptosporidium* spp. in Qinghai province was found to be the highest among the analyzed provinces. Several studies showed that the prevalence of *Cryptosporidium* spp. in other animals was also at a high level in Qinghai province. For instance, the prevalence of *Cryptosporidium* spp. is identified to be 22.8% and 39.02% in sheep and goats, respectively (Karanis et al., 2007; Niu and Ma, 2007; Ma et al., 2010; Ma et al., 2013). Some of the water in Qinghai province contains high concentration of *Cryptosporidium* spp. oocysts (Ma et al., 2014a; Ma et al., 2019), and the infected animals were also potential factors inducing water pollution. The oocysts in the environment were difficult to be eliminated, thus resulting in an increased *Cryptosporidium* spp. infection rate in yaks through ingesting contaminated water (Li et al., 2016b; Li et al., 2019). This may lead to an increase of *Cryptosporidium* spp. infection in yaks. Multiple factors, such as climate change, animal husbandry practices, and parasite control measures, may cause various prevalence in different geographic regions (Taghipour et al., 2020). The latitude and longitude of Qinghai province are “31°36’-99°19’” and “89°35’-103°04’”, respectively. At the same time, we found that the areas with latitude > 35° and longitude of 95-100° were also located in Qinghai province, and the infection rate was high (**Table 4**). Qinghai province has a typical continental plateau climate (Zhang, 2010) that is high altitude, low temperature, and unpredictable climate (Wei et al., 2015; Zhang et al., 2019). The same characteristics were also observed in our climate subgroup analysis. The prevalence of *Cryptosporidium* spp. in the continental plateau climate was higher than that in other subgroups.

The prevalence of *Cryptosporidium* spp. in yaks < 12 months was higher than that ≥12 months based on our data. The age of sexual maturity of the yak is about 12 months, so the age “12 months” is taken as the cut-off age-point (Wen, 1988). The maternal antibodies obtained from colostrum in young yaks disappear approximately in 2-6 months, therefore, the immunity may decrease and then result in an increased morbidity (Sareyyüpoğlu et al., 2019; Wang et al., 2020). The prevalence of *Cryptosporidium* spp. was slightly lower in the younger yaks.

To date, a total of 12 *Cryptosporidium* spp. species/genotypes were identified in yaks. Among these species/genotypes, *C. parvum*, *C. hominis*, and *C. ubiquitum* were identified in humans, which has caused a widespread concern (Widmer, 2009; Li et al., 2014; Ryan et al., 2016). Interestingly, co-infection of two species/genotypes (*C. ryanae* and *C. bovis* or *C. parvum* and *C. bovis*) was also found in yaks (Mi et al., 2013; Ma et al., 2014b), suggesting that the environment might be contaminated by more than one *Cryptosporidium* spp. species/genotype. The present study found that *C. bovis* had the highest prevalence in the investigated yaks. *C. bovis* is one of the main genotypes that cause cryptosporidiosis in cattle (Wang et al., 2017) and *C. bovis* has been found to be the most prevalent species in pre-weaned calves (Wang et al., 2011; Murakoshi et al., 2012; Zhang et al., 2013). Other studies have also confirmed *C. bovis* was the dominant species in cattle (Mi et al., 2013; Ma et al., 2014b).

In the subgroup of precipitation, the prevalence of *Cryptosporidium* spp. at altitude < 3000 m was higher than that at altitude > 3000 m. Additionally, the temperature was usually high at the low altitude. Previous studies showed that cryptosporidiosis mainly occurred in warm and humid seasons (Lou, 2016). In the subgroups of precipitation and humidity, we found that the prevalence of *Cryptosporidium* spp. in precipitation (> 300 mm) and humidity (> 55%) environment was also high. Thus, our data were in line with previous findings (Taghipour et al., 2020).

The prevalence of *Cryptosporidium* spp. in the cold weather was higher than that in the warm weather, owing to a generally lower temperature on the plateau (Taghipour et al., 2020). Due to the special physiological characteristics of the yak, most of the yaks are grazing in the resource-rich plateau grasslands (Fu et al., 2018). The forage has a low nutrient content in the cold weather, which does not meet the nutrients required by yaks. This causes a loss of body weight and a decreased immunity of yaks, and thus increasing the probability of *Cryptosporidium* spp. infection and prevalence. The forage becomes enriched after the end of cold weather. The body weight and resistance of yaks will increase in the warm weather (Zhou et al., 2020). This may be the reason for the lowest prevalence of *Cryptosporidium* spp. observed in the seasons with a lower temperature. Thus, we suggest an increased feed should be provided in time to enhance the resistance of yaks in the cold weather.

In this study, the prevalence of *Cryptosporidium* spp. with Enzyme-linked immunosorbent assay (ELISA) was higher than that with the other three methods in previous reports. ELISA has high specificity and large sample size (Liu et al., 2015; Gong et al., 2020). However, ELISA cannot be used for species typing. In addition, ELISA was rarely used to detect species of parasites (Seema et al., 2014). In this subgroup, there were fewer articles using ELISA to detect *Cryptosporidium* spp., and the lack of data in this subgroup might lead to a higher prevalence than the other groups, thus resulting in unstable results. The advantages of microscope inspection include simple operation, reasonable price, and easy to capture (Taghipour et al., 2020). Microscopic examination can be used to detect intestinal parasitic infection and shows the presence of pathogens and non-pathogenic parasites, but the specific detection of different *Cryptosporidium* spp. species is not reliable (Incani et al., 2017; Taghipour et al., 2020). Microscopy also has a low sensitivity which may lead to false positive (Wang et al., 2020). This may be one reason for the high prevalence. IFA has high sensitivity, specificity, and stability for detection of oocysts. The sensitivity is high for even a low oocyst concentration (Ahmed and Panagiotis, 2018). A cross-reaction with fecal yeast during a longer treatment process is one of disadvantages for IFA (Johnston et al., 2003). PCR allows a simultaneous detection of different parasites in a single reaction, which has a higher sensitivity and easier interpretation (Incani et al., 2017). PCR can be used to detect complete DNA and fragments of parasites, and has become the best method for detecting *Cryptosporidium* spp. (Efrat et al., 2019). Thus, we suggest that the researchers to use the PCR method for detecting *Cryptosporidium* spp. during epidemiological investigations.

In our meta-analysis (n = 20), there are 5 medium-quality articles and 1 low-quality article. The reason for appearance of medium- or low-quality articles was that most studies had a sample size less than 200 and less than 3 risk factors. It is recommended that researchers should take a large sample size, explore more risk factors, clarify the cause of *Cryptosporidium* spp. infection, and provide scientific data and theoretical support for the prevention and control of *Cryptosporidium* spp. infection in yaks.

There were several limitations for our meta-analysis. First, the studies from five databases were limited for obtaining all relevant research data. Second, most of the data were derived from Qinghai province, leading to an uneven data distribution in the northwestern China, and thus affecting the true positive rate. Third, since most of the data show that the yaks are free-range, there is no way to analyze the impact of the feeding mode on *Cryptosporidium* spp. Finally, the available data for this analysis are limited.

## Conclusions

The results of this systematic review and meta-analysis using 20 articles showed that *Cryptosporidium* spp. is common in yaks in China. Different seasons and sampling years had a statistically significant effect on the *Cryptosporidium* spp. infection in yaks. Yaks under 12 months had a higher prevalence of *Cryptosporidium* spp. Thus, the protective measures should be strengthened at this age stage. This study provided basic data for the prevention and control of cryptosporidiosis in yaks. This may help monitor the prevalence of *Cryptosporidium* spp. in yaks, prevent and control *Cryptosporidium* spp. infection in yaks, in order to reduce the risk of *Cryptosporidium* spp. infection in humans.

## Data Availability Statement

The original contributions presented in the study are included in the article/**Supplementary Material**. Further inquiries can be directed to the corresponding authors.

## Ethics Statement

The data regarding the Yaks were collected from five online databases (Chinese National Knowledge Infrastructure (CNKI), VIP Chinese journal database, WanFang Data, PubMed, and ScienceDirect). Written informed consent was obtained from the owners for the participation of their animals in this study.

## Author Contributions

H-TS, JJ, and H-BN were responsible for the idea and concept of the paper. X-YW and WW built the database. H-LG and WW analyzed the data. H-LG wrote the manuscript. J-HL, JJ, and X-YW critically reviewed and revised the manuscript. All authors contributed to the article and approved the submitted version.

## Funding

This work was supported by the Wild Animal Disease Monitoring and Early Warning System Maintenance Project (2130211), and the Research Foundation for Distinguished Scholars of Qingdao Agricultural University (665-1120046).

## Conflict of Interest

The authors declare that the research was conducted in the absence of any commercial or financial relationships that could be construed as a potential conflict of interest.

## Publisher’s Note

All claims expressed in this article are solely those of the authors and do not necessarily represent those of their affiliated organizations, or those of the publisher, the editors and the reviewers. Any product that may be evaluated in this article, or claim that may be made by its manufacturer, is not guaranteed or endorsed by the publisher.

## Abbreviations

VIP, VIP Chinese Journal Databases; CNKI, China National Knowledge Infrastructure; WanFang, WanFang Databases.
