# Supplementary material for: Prevalence of Cryptosporidium spp. in Yaks (Bos grunniens) in China: A Systematic Review and Meta-Analysis
Source: Front Cell Infect Microbiol. 2021 Oct 18;11:770612. doi: 10.3389/fcimb.2021.770612 (PMC8558464; doi:10.3389/fcimb.2021.770612)
Supplement: Supplementary file 1 [file Table_1.docx]

**Table S1** Studies included in the analysis.

| **Study ID** | **Sampling time** | **Province** | **Method** | **No. Positive/**  **No. Tested** | **Quality score** | **Study Quality** |
| --- | --- | --- | --- | --- | --- | --- |
| Wang (2018) | 2016.06-2017.09 | Qinghai | PCR | 26/1027 | 4 | High |
| Li (2016) | 2013.06-2015.05 | Qinghai | PCR | 158/554 | 4 | High |
| Hao et al. (2016) | UN* | Sichuan | PCR | 32/216 | 3 | High |
| Mi et al. (2013) | 2008.03-2012.06 | Qinghai | Microscopy | 142/586 | 4 | High |
| Wang et al. (2007) | 2006.05-2006.06 | Qinghai | Microscopy | 32/190 | 3 | High |
| Ma et al. (2011) | 2009.05-2009.07 | Qinghai | ELISA | 368/1094 | 3 | High |
| Wang et al. (2018) | 2015-2016 | Qinghai | PCR | 39/344 | 3 | High |
| Wang et al. (2018) | 2015-2016 | Qinghai | Microscopy | 15/344 | 3 | High |
| Wang et al. (2018) | 2015-2016 | Qinghai | IFA | 40/344 | 3 | High |
| Li et al. (2020b) | 2019 | Tibet | PCR | 103/950 | 4 | High |
| Qi et al. (2015) | 2009-2012 | Tibet, Gansu, Sichuan and Qinghai | PCR | 22/545 | 4 | High |
| Ma et al. (2014) | 2013.05-2013.06 | Qinghai | PCR | 98/327 | 4 | High |
| Wang (2014) | 2013.07-2013.11 | Sichuan | Microscopy | 0/261 | 3 | High |
| Bai et al. (2001) | 1996.05 | Qinghai | Microscopy | 26/80 | 3 | High |
| Mu (2018) | 2016.06-2016.07 | Tibet | PCR | 8/577 | 4 | High |
| Du et al. (2020) | 2019.7-2019.10 | Qinghai | PCR | 2/200 | 4 | High |
| Du et al. (2020) | 2019.7-2019.10 | Qinghai | IFA | 3/200 | 4 | High |
| Zhou et al. (2009) | UN* | Qinghai and Shanghai | Microscopy | 13/402 | 1 | Low |
| Zhang et al. (2006) | 2004.08; 2005.03 | Qinghai | Microscopy | 60/151 | 2 | Middle |
| Zhou (2009) | UN* | Qinghai | Microscopy | 60/456 | 2 | Middle |
| Tie et al. (2011) | 2009.06-2009.09 | Qinghai | ELISA | 1/135 | 2 | Middle |
| Ma et al. (2013) | 2005.09-2011.09 | Qinghai | PCR | 0/16 | 2 | Middle |
| Ma et al. (2013) | 2005.09-2011.09 | Qinghai | IFA | 2/16 | 2 | Middle |
| Zhang et al. (2019) | 2018.05-2019.10 | Qinghai, Yunnan and Tibet | PCR | 2/101 | 2 | Middle |

UN*: unclear.

**References**

Bai, Y.H., Suo, N.D.J., Jia, G.Y., Xing, S.J., Ma, Z.Y., Zeng, B. (2001). Investigation of Cryptosporidiosis in Yak Calves in Tongde County. Sichuan Anim Vet Scis. 2001:22. (in Chinese)

Du, M.Z., Zhang, C.D.Z.M., Zhao, Z.G., Gu, D.H., Li, G.P., Yi, P.C. (2020). Investigation on *Cryptosporidium* Infection and Species Identification of Resident Yak in Datong County, Qinghai Province. Anim Husband Feed Sci. 41:106-110. (in Chinese)

Hao, L.L., Li, R., Duan, L., He, L. (2016). Molecular Epidemiology of *Cryptosporidium* Infection in Yaks in Hongyuan County, Sichuan Province. Acta Agricul Zhejiang. 28:1842-1846. (in Chinese)

Li, P. (2016). Distribution and Public Health Significance of *Cryptosporidium* and *Enterocytozoon Bieneusi* in Tibetan Sheep and Yaks in Qinghai Province. East China Univ Sci Technol. 2016:74. (in Chinese)

Li, K., Li, Z., Zeng, Z., Li, A., Mehmood, K., Shahzad, M., et al. (2020b). Prevalence and Molecular Characterization of *Cryptosporidium* Spp. in Yaks (*Bos Grunniens*) in Naqu, China. Microb Pathog. 144:104190. doi: 10.1016/j.micpath.2020.104190

Ma, L.Q., Lu, Y., Cai, Q.G., Wang, G.P., Niu, X.Y., Ye, C.Y., et al. (2011). Serological Investigation of Cryptosporidiosis in Yaks in Qinghai Province. J Domest Anim Ecol. 32:47-49. (in Chinese)

Ma, L.Q., Wang, G.P., Lu, Y., Cai, Q.G., Wan,g G.H., Li, X.P., et al. (2013). Molecular Characteristics of *Cryptosporidium* in Qinghai Province. Chinese Qinghai J Anim Vet Sci. 2013:1-3. (in Chinese)

Mi, R., Wang, X., Li, C., Huang, Y., Zhou, P., Li, Z., Lei, M., et al. (2013). Prevalence and Genetic Characterization of *Cryptosporidium* in Yaks in Qinghai Province of China. PLoS One. 8(9):e74985. doi: 10.1371/journal.pone.0074985

Ma, J., Cai, J., Ma, J., Feng, Y., Xiao, L. (2014). Occurrence and Molecular Characterization of *Cryptosporidium* Spp. in Yaks (*Bos Grunniens*) in China. Vet Parasito. 202(3-4):113-8. doi: 10.1016/j.vetpar.2014.03.030

Mu, Y.Y. (2018). Molecular Epidemiology and Zoonotic Risk Analysis of Three Intestinal Protozoa in Yaks in Tibet. Henan Agr Univ. 2018:65. (in Chinese)

Qi, M., Cai, J., Wang, R., Li, J., Jian, F., Huang, J., et al. (2015). Molecular Characterization of *Cryptosporidium* Spp. and *Giardia Duodenalis* from Yaks in the Central Western Region of China. BMC Microbiol. 15:108. doi: 10.1186/s12866-015-0446-0

Tie, F.P., Li, D.S. (2011). Investigation on the Causes of Abortion of Yak in Haiyan County, Qinghai Province. Chin J Anim Sci. 47:50-51. (in Chinese)

Wang, C.J., Liu, H.M. (2007). Epidemiological Investigation of *Cryptosporidium* in Yak in Hualong County of Qinghai Province. Chin J Anim Sci. 2007: 31. (in Chinese)

Wang, F.Y. (2014). Study on the Development of *Cryptosporidium* from Tibetan sheep, Rabbit and Cattle in Some Areas of Sichuan Province. Sichuan Agr Univ. 2014:7.1 (in Chinese)

Wang, D. (2018). Prevalence and Population Structure of *Cryptosporidium* And *Giardia Lamblia* in Yaks in Qinghai Province. Northwest AF Univ. 2018:62. (in Chinese)

Wang, G., Wang, G., Li, X., Zhang, X., Karanis, G., Jian, Y., et al. (2018). Prevalence and Molecular Characterization of *Cryptosporidium* Spp. and *Giardia Duodenalis* in 1-2-Month-Old Highland Yaks in Qinghai Province, China. Parasito Res. 117:1793-1800. doi: 10.1007/s00436-018-5861-3

Zhang, J., Xu, J.F., Shen, X.Y. (2006). Investigation on the Infection of *Cryptosporidium Bovis* in Qinghai Province. Chin Qinghai J Anim Vet Sci. 2006:16-17. (in Chinese)

Zhou, C.X. (2009). Investigation and Molecular Identification of *Cryptosporidium* Infection in Yak and Cynomolgus Monkey. Henan Agr Univ. 2009:51. (in Chinese)

Zhou, C.X., He, G.S., Zhang, L.X. (2009). Investigation of *Cryptosporidium* Infection in Yak. Chin J Zoonoses. 25:389-390. (in Chinese)

Zhang, Q., Zhan,g Z., Ai, S., Wang, X., Zhang, R., Duan, Z. (2019). *Cryptosporidium* spp., *Enterocytozoon Bieneusi*, And *Giardia Duodenalis* from Animal Sources in the Qinghai-Tibetan Plateau Area (QTPA) in China. Comp Immunol Microbiol Infect Dis. 67:101346. doi: 10.1016/j.cimid.2019.101346
